# Optimizing Antihypertensive Medication Classification in Electronic Health Record-Based Data: Classification System Development and Methodological Comparison

**DOI:** 10.2196/14777

**Published:** 2020-02-27

**Authors:** Caitrin W McDonough, Steven M Smith, Rhonda M Cooper-DeHoff, William R Hogan

**Affiliations:** 1 Department of Pharmacotherapy and Translational Research College of Pharmacy University of Florida Gainesville, FL United States; 2 Division of Cardiovascular Medicine, Department of Medicine College of Medicine University of Florida Gainesville, FL United States; 3 Department of Health Outcomes & Biomedical Informatics College of Medicine University of Florida Gainesville, FL United States

**Keywords:** antihypertensive agents, electronic health records, classification, RxNorm, phenotype

## Abstract

**Background:**

Computable phenotypes have the ability to utilize data within the electronic health record (EHR) to identify patients with certain characteristics. Many computable phenotypes rely on multiple types of data within the EHR including prescription drug information. Hypertension (HTN)-related computable phenotypes are particularly dependent on the correct classification of antihypertensive prescription drug information, as well as corresponding diagnoses and blood pressure information.

**Objective:**

This study aimed to create an antihypertensive drug classification system to be utilized with EHR-based data as part of HTN-related computable phenotypes.

**Methods:**

We compared 4 different antihypertensive drug classification systems based off of 4 different methodologies and terminologies, including 3 RxNorm Concept Unique Identifier (RxCUI)–based classifications and 1 medication name–based classification. The RxCUI-based classifications utilized data from (1) the Drug Ontology, (2) the new Medication Reference Terminology, and (3) the Anatomical Therapeutic Chemical Classification System and DrugBank, whereas the medication name–based classification relied on antihypertensive drug names. Each classification system was applied to EHR-based prescription drug data from hypertensive patients in the OneFlorida Data Trust.

**Results:**

There were 13,627 unique RxCUIs and 8025 unique medication names from the 13,879,046 prescriptions. We observed a broad overlap between the 4 methods, with 84.1% (691/822) to 95.3% (695/729) of terms overlapping pairwise between the different classification methods. Key differences arose from drug products with multiple dosage forms, drug products with an indication of benign prostatic hyperplasia, drug products that contain more than 1 ingredient (combination products), and terms within the classification systems corresponding to retired or obsolete RxCUIs.

**Conclusions:**

In total, 2 antihypertensive drug classifications were constructed, one based on RxCUIs and one based on medication name, that can be used in future computable phenotypes that require antihypertensive drug classifications.

## Introduction

### Background

Electronic health records (EHRs) contain a wealth of clinical information, and the development of tools to perform structured queries of common data models (CDMs) to standardized data formats has allowed researchers to utilize EHR data for discriminating complex clinical phenotypes with high measurement validity [1-4]. Measuring such complex phenotypes often requires integration of multiple streams of EHR data, including diagnoses or procedures, clinical measurements (eg, vitals and laboratory parameters), and medications or other exposures [5]. Additionally, the accurate measurement of these complex phenotypes relies on accurate measurement and classification of these individual components [6,7]. The classification of medication use in this context offers unique challenges because many medications have multiple indications and dosage forms. Furthermore, existing therapeutic classification systems generally group drug entities in ways that may only partially correlate with intended use [8], eg, numerous medications block beta-adrenergic receptors (beta-blockers), and such an effect can lower blood pressure (BP), but not all beta-blockers are used clinically as antihypertensives. Even among beta-blockers that are used clinically as antihypertensives, not all dosage forms effectively treat systemic hypertension (HTN) [9]. Thus, the mere presence of a drug entity in the prescribing record may not be sufficient to meet medication-related criteria in a complex phenotype.

### Objectives

Accordingly, we aimed to design a set of standardized antihypertensive drug codes to be usable and maintainable by both ourselves and others in the research community [10]. We performed this study in the context of optimizing medication classification for use in HTN-related computable phenotypes. To properly identify patients who are taking antihypertensive medications, the prescription drug information from the EHR must be properly classified into antihypertensive therapeutic indication and antihypertensive drug classes. We designed our study by utilizing concepts (RxNorm and medication name) present in the prescription drug information from data models common in the United States (Informatics for Integrating Biology & the Bedside [i2b2], The National Patient Centered Clinical Research Network [PCORnet] CDM, and The Observational Medical Outcomes Partnership [OMOP] CDM) [1,3,4]. The main objective of this study was to report the development of a set of standardized drug codes and names for use in querying EHR data for antihypertensive medication prescriptions. The second objective was to compare different methods and resources for creating the antihypertensive drug classification. The third and final objective was to explore the coverage of different methods and resources (for creating the classification) on querying a large EHR-based dataset.

## Methods

### Resources

#### RxNorm

RxNorm is a standardized terminology to represent drugs. It was developed by the US National Library of Medicine (NLM) in 2002 and represents medications through normalized names for clinical drugs, which include ingredient or ingredients strength or strengths, and dose form [11,12]. The normalized names allow equivalent drug terms from different source vocabularies to be grouped together under the same RxNorm Concept Unique Identifier (RxCUI) [11]. RxNorm was designed to support electronic prescribing, mapping between different drug vocabularies, and the development of medication-related clinical decision support rules [11,12]. RxNorm can be accessed through an application programming interface (API), the RxNorm Navigator (RxNav) [12-14]. Currently, RxNorm integrates drug terminology from many sources including most drug knowledge bases (eg, Multum, Micromedex, and First DataBank), standard terminologies (eg, SNOMED CT and MeSH), the new Medication Reference Terminology (MED-RT), federal agencies in the United States (eg, Food and Drug Administration Structured Product labels), and international drug resources such as the Anatomical Therapeutic Chemical (ATC) Classification System and DrugBank [11,15].

#### Drug Ontology

The Drug Ontology (DrOn) is a formal representation of drug products, drug ingredients, mechanisms of action, therapeutic indications, strengths, and dosage forms based on the OWL2 Web Ontology Language [8,16-18]. These representations were created to allow researchers to query drug datasets, which usually come from EHRs or health insurance claim databases and typically use RxCUIs to identify different aspects of drug products (ie, ingredient or ingredients, dose forms, and strength or strengths), and National Drug Codes to identify individual drug products and the manufacturer and packaging thereof [16,17]. Currently, DrOn represents a drug’s therapeutic indication as being a property of a drug product, which is a composite of one or more drug ingredients and excipients, whereas its mechanism of action (MoA) is represented as a property of the chemical compound or chemical compounds that constitutes the drug’s ingredients [8,16,17]. DrOn has a hand-curated component and a component built automatically from RxNorm [16-18]. At present, DrOn contains representations for several mechanisms of action belonging to various antihypertensive drugs classes [8,16,17]. It also contains class representations for the drug products and ingredients, dosage forms, strengths, and therapeutic indications.

### Data Source

#### OneFlorida and the OneFlorida Hypertension Population

The OneFlorida Clinical Research Consortium is a statewide network of health systems, providers, and payers covering more than 74% of Florida’s population [19]. The network’s catchment area covers all 67 Florida counties and allows for the facilitation of clinical and translational research in health care settings and communities throughout the state. OneFlorida houses a Data Trust, which contains longitudinal EHR data on approximately 14 million Floridians (approximately 66%), mapped to the PCORnet CDM [19,20]. The HTN population within OneFlorida was defined as all adults (aged ≥18 years) with ≥1 HTN diagnosis from an outpatient encounter, defined as International Classification of Diseases (ICD)-9 code 401.x (Essential HTN) or ICD-10 code I10 (Essential [primary] HTN). The data utilized for this study were extracted from the OneFlorida Data Trust on December 14, 2017, in the PCORnet CDM, version 3.0, and included EHR data from June 2000 to July 2017, with 99.97% from encounters occurring from January 2011 onward. All (100%) of the prescription drug data were from January 2011 onward.

#### Prescription Drug Data

All prescription drug data for the HTN population were extracted from the Prescribing Table, including information on raw medication name and RxCUI. A total of 2 drug lists were created from this dataset. The first contained all unique raw medication names (see Drug Classification by Ingredient Name) derived directly from source EHRs, ie, not cleaned or curated during mapping to the PCORnet CDM. The second contained all unique RxCUIs (see Drug Classification by RxCUI utilizing DrOn and Drug Classification by RxCUI utilizing RxClass), which may be derived from source EHRs or created during mapping to the PCORnet CDM.

### Drug Classification by Ingredient Name

A Medication Name Classification was constructed for antihypertensive medications, utilizing drug ingredient names. A summary of the features included is available in Multimedia Appendix 1. Both brand name and generic name were included. The list was constructed through manual curation, including methods from prior antihypertensive drug classifications [21,22]. The manually curated list was also reviewed by authors with biomedical informatics expertise (CM) and HTN pharmacotherapy expertise (CM and RC). The Medication Name Classification contains 286 drug ingredient names, further classified by antihypertensive drug class (eg, beta-blockers, angiotensin II receptor inhibitors [ARBs], calcium channel blockers [CCBs], etc).

To apply the Medication Name Classification to the OneFlorida raw medication list, the first word in the field was extracted as the ingredient name, with additional coding to capture combination medications (eg, adding underscores between the first and second words) and strings where the ingredient was not the first word. The antihypertensive Medication Name Classification was merged with the unique raw medication names from the OneFlorida dataset to map the drugs by antihypertensive drug class. All of the raw medication names that did not merge with the Medication Name Classification were discarded (eg, statins, insulin, etc).

### Drug Classification by RxNorm Concept Unique Identifier Utilizing the Drug Ontology

The DrOn RxCUI Classification was constructed through multiple steps (Multimedia Appendix 1). First, all RxCUIs with a therapeutic indication for HTN (antihypertensive function) were extracted from DrOn using the *dron-query* tool [8]. Then, separate lists of RxCUIs were extracted for drugs with any of the following mechanisms of action: angiotensin-converting enzyme (ACE)–inhibitor, ARB, beta-blocker, CCB, loop diuretic, and thiazide and thiazide-like diuretic. These lists were then merged to assign an antihypertensive MoA to each RxCUI with a therapeutic indication for HTN. Combination drug products were assigned multiple mechanisms of action, representing each ingredient. The list was then manually reviewed, and mechanisms of action were added for drug products with mechanisms of action not currently represented in DrOn. These included the following: aldosterone antagonists, direct renin inhibitors, alpha-1 blockers, potassium-sparing diuretics, vasodilators, centrally acting agents, and other agents. The list was then reviewed by authors with biomedical informatics expertise (CM and WH) and HTN pharmacotherapy expertise (CM, SS, and RC). The DrOn RxCUI Classification contains 2543 antihypertensive RxCUIs, of SCDF, SCD, and SBD term types, organized by antihypertensive drug class or drug classes.

The unique RxCUIs from the OneFlorida dataset were merged with the DrOn RxCUI Classification to map the drugs by antihypertensive drug class. All of the RxCUIs that did not merge with the DrOn RxCUI Classification were discarded (eg, statins, insulin, etc).

### Drug Classification by RxNorm Concept Unique Identifier Utilizing RxClass

The unique list of RxCUIs extracted from OneFlorida were also mapped utilizing the RxClass API on RxMix [23]. RxCUIs with less than 4 digits were removed (n=25). The function, “getClassByRxNormDrugId,” was used to obtain the drug classes for a specified drug identifier. In total, 2 different relationship sources (relaSource) were tested: ATC and MED-RT (Multimedia Appendix 1). Within MED-RT, the following relationships (rela) were selected: “has_MoA” and “may_treat.” The unique RxCUIs from the OneFlorida dataset were classified using the ATC and MED-RT relationship sources through the batch input mode.

### Comparison Between Drug Classifications

The different drug classification methods were compared pairwise by calculating percent coverage and by reviewing the overlapping and nonoverlapping sets of RxCUIs among them. For all classification methods, the percent of antihypertensive drugs covered was calculated as the number of antihypertensive medications mapped by the classification method divided by the total number of unique terms (Raw Name or RxCUI) contained in the OneFlorida Prescribing Table. Within the ATC relationship source from RxClass, the antihypertensive classes were selected from the “name” field. A complete list of the ATC relationships included as antihypertensive drugs is available in Multimedia Appendix 2. Within the MED-RT relationship source from RxClass, 2 steps were used to select antihypertensive drugs: (1) RxCUIs were selected with the “may_treat” HTN relationship and (2) RxCUIs were further filtered based on the “has_MoA” relationship, including only those drugs from antihypertensive classes. A complete list of the MED-RT “has_MoA” relationships included as antihypertensive drugs is available in Multimedia Appendix 3. Within the MED-RT relationship source from RxClass, the results from “may_treat” relationship were used to calculate total coverage. Differences between the antihypertensive coverage results were identified by pairwise comparisons and merges between the DrOn RxCUI Classification and every other classification.

On the basis of our review of the overlapping and nonoverlapping sets of names and RxCUIs among the classifications, we created a version 1.0 of 2 finalized classifications—one for ingredient names and one for RxCUIs—for use by us and other researchers. We published them on GitHub [10] to make them findable and reusable and to enable community contributions to future versions.

### Application of the Drug Classifications to OneFlorida

The Medication Name Classification and the DrOn RxCUI Classification were applied to all of the prescription drug data from the OneFlorida HTN population. All available prescriptions from January 2011 onward were considered. Antihypertensive coverage by each method was calculated as number of prescription records mapped to an antihypertensive drug class by each map divided by the total number of prescriptions. Differences between the coverage results were identified by pairwise comparison. Additionally, summary-level frequency counts and percentages by the antihypertensive drug class were also calculated. All coding for mapping the drug data and the summary statistics was conducted using SAS version 9.4 (SAS).

## Results

### Data Source

At the time of the data extraction, there were 1,188,977 patients in the OneFlorida Data Trust with an ICD-9 or ICD-10 diagnosis code for HTN (Table 1).

In total, there were 13,879,046 prescriptions from these patients over the study period (January 2011 to July 2017; approximately 6.6 years). These prescriptions consisted of 13,627 unique RxCUIs and 8025 unique first words from the raw medication name string (Table 1).

**Table 1:** Counts of data attributes in the OneFlorida hypertensive patient prescribing table dataset.

Data attributes	Values, N
Hypertensive patients	1,188,977
Prescription records	13,879,046
Unique RxCUIs^a^	13,627
Unique Raw Med Name^b^	8025

^a^RxCUI: RxNorm Concept Unique Identifier.

^b^Unique first word of the Raw_Med_Name field, after additional data cleaning and quality control steps.

### Drug Classification by Ingredient Name

The initial Medication Name Classification contained 286 antihypertensive medications that are mapped to 35 antihypertensive medication classes or combination medication classes (eg, ACE inhibitors, ARBs, CCBs, CCB-ARB combinations, etc). We chose to exclude timolol to be conservative. On the basis of this classification system, it is impossible to distinguish between oral and ophthalmic products. An excerpt from the Medication Name Classification is shown in Table 2, whereas the full map is available in Multimedia Appendix 4. Each entry consists of an arbitrary code, the single word drug name, the generic name (if applicable), and the drug class.

**Table 2:** Excerpt from Medication Name Classification.

Code^a^	Drug_Name	Generic^b^	Drug_Class
1	Benazepril	—^c^	ACE^d^
2	Lotensin	benazepril	ACE
3	Captopril	—	ACE
4	Capoten	captopril	ACE
5	Enalapril	—	ACE
6	Enalaprilat	enalapril	ACE
7	Fosinopril	—	ACE
8	Monopril	fosinopril	ACE
9	Lisinopril	—	ACE
10	Prinivil	lisinopril	ACE
11	Zestril	lisinopril	ACE

^a^Full classification list available in Multimedia Appendix 4.

^b^Generic drug name included for brand name drugs.

^c^Not applicable for generic drugs.

^d^ACE: angiotensin-converting enzyme inhibitor.

### Drug Classification by RxNorm Concept Unique Identifier Utilizing the Drug Ontology

The initial DrOn RxCUI Classification contained 2543 antihypertensive RxCUIs that were mapped to 46 antihypertensive medication classes or combination medication classes. Each RxCUI entry contains the RxCUI, the drug product, and the drug class. An excerpt of the DrOn RxCUI Classification is shown in Table 3, and the full map is available in Multimedia Appendix 5.

**Table 3:** Excerpt from the Drug Ontology RxNorm Concept Unique Identifier classification.

RxCUI^a,b^	Drug_Product	Rx_Norm_Drug_Class
858926	Enalapril Maleate 20 MG Chewable Tablet	ACE^c^
378269	Enalapril Chewable Tablet	ACE
858810	Enalapril Maleate 20 MG Oral Tablet	ACE
858804	Enalapril Maleate 2.5 MG Oral Tablet	ACE
858821	Enalapril Maleate 1.25 MG/ML Injectable Solution	ACE
858817	Enalapril Maleate 10 MG Oral Tablet	ACE
858813	Enalapril Maleate 5 MG Oral Tablet	ACE
372007	Enalapril Oral Tablet	ACE
378288	Enalapril Injectable Solution	ACE
246264	Enalaprilat 1 MG/ML Injectable Solution	ACE
374378	Enalaprilat Injectable Solution	ACE
252820	Enalaprilat 0.625 MG/ML Injectable Solution	ACE
204404	Enalaprilat 1.25 MG/ML Injectable Solution	ACE

^a^Full map available in Multimedia Appendix 5.

^b^RxCUI: RxNorm Concept Unique Identifier.

^c^ACE: angiotensin-converting enzyme inhibitor.

### Comparison Between Drug Classifications

The percent of drugs covered by each antihypertensive map is shown in Table 4. Using the Medication Name Classification, 207 ingredient terms were mapped to antihypertensive drug classes out of a total of 8025 unique raw medication names (2.58%; Table 4). When the DrOn RxCUI Classification was applied to the unique RxCUIs (n=13,627), 729 were successfully mapped to antihypertensive drug classes (5.35%; Table 4). Through the RxClass API, using ATC as the relationship source, 822 out of 13,602 RxCUIs were mapped to antihypertensive drug classes (6.04%; Table 4). Finally, when the RxClass API was used with MED-RT as the relationship source, 792 RxCUIs had the relationship of “may_treat” HTN and a “has_MoA” that corresponded to an antihypertensive drug class (792/13,602, 5.82%; Table 4).

**Figure 1:**
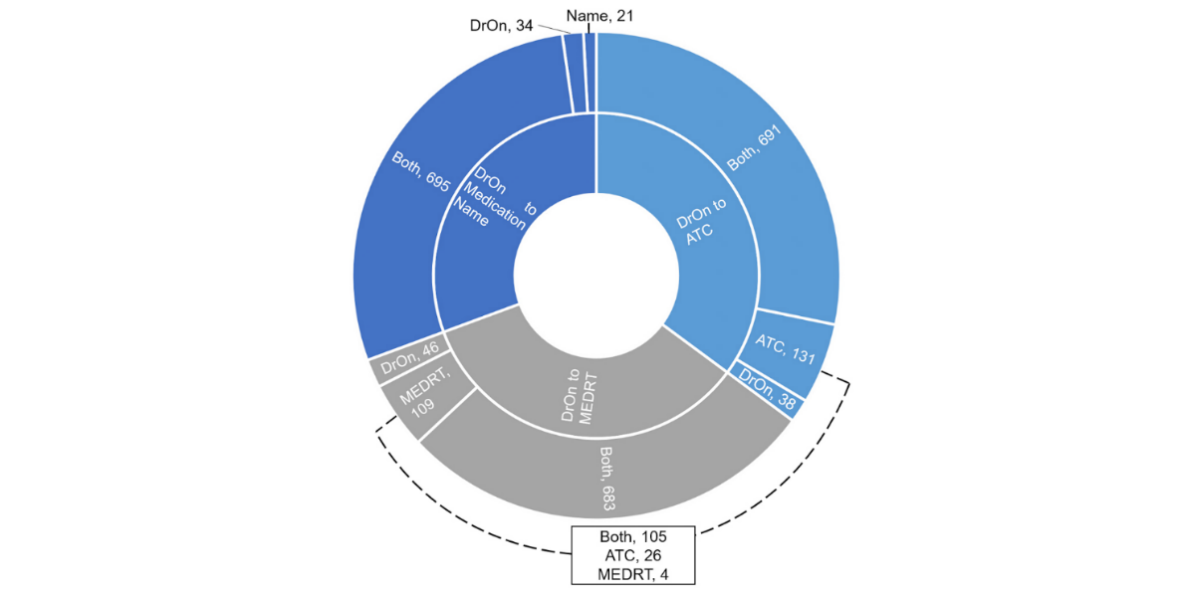
Comparisons of the Drug Ontology RxNorm Concept Unique Identifier Classification to the other classification methods. Results are shown for the number overlapping between the methods (Both) and the numbers unique to each method. ATC: Anatomical Therapeutic Chemical; MED-RT: Medication Reference Terminology.

When the DrOn RxCUI Classification was compared with the other classifications, they broadly overlapped; however, there were some key differences (Figure 1). When the DrOn RxCUI Classification was compared with the other RxCUI-based maps constructed through the RxClass API, 691 terms overlapped with the RxClass-ATC map and 683 terms overlapped with the RxClass–MED-RT map. There were 38 terms and 46 terms that were unique to the DrOn RxCUI map when compared with the RxClass-ATC map and RxClass–MED-RT map, respectively. Additionally, there were 131 terms that were unique to the RxClass-ATC map, and 109 terms that were unique to the RxClass–MED-RT map. Of these 131 terms and 109 terms, respectively, there were 135 unique terms, with 105 overlapping between the RxClass-ATC map and the RxClass–MED-RT map.

Of the 135 unique RxCUIs from the RxClass ATC and MED-RT maps, 29 had a term type of *IN*, *MIN*, or *PIN*, representing antihypertensive medications. These term types were not included in the DrOn RxCUI map owing to their low level of specificity. Next, there were 24 RxCUIs that were either ophthalmic or topical drug products. Examples of these are shown in Table 5.

**Table 4:** Drug coverage by each classification method.

Classification method	Input term	Input, N	Antihypertensive	
			Mapped, n	Coverage, %
Medication Name	Raw Name	8025	207	2.58
DrOn^a^ RxCUI^b^	RxCUI	13,627	729	5.35
RxClass-ATC^c^	RxCUI	13,602	822	6.04
RxClass–MED-RT^d,e^	RXCUI	13,602	792	5.82

^a^DrOn: Drug Ontology.

^b^RxCUI: RxNorm Concept Unique Identifier.

^c^ATC: Anatomical Therapeutic Chemical.

^d^MED-RT Antihypertensive coverage was mapped using 2 steps: (1) "may_treat" hypertension and (2) mechanism of action of an antihypertensive drug class.

^e^MED-RT: Medication Reference Terminology.

**Table 5:** Examples of RxNorm Concept Unique Identifiers inappropriately mapped as antihypertensives utilizing the RxMix tools.

RxCUI^a^	DrugName	hasMOA	ConceptName	DoseForm
207371	Minoxidil	Potassium Channel Interactions	Minoxidil 20 MG/ML Topical Solution (Rogaine)	Topical solution
208560	Timolol	Adrenergic beta1-Antagonists	Timolol 2.5 MG/ML Ophthalmic Solution (Betimol)	Ophthalmic solution
213729	Betaxolol	Adrenergic beta1-Antagonists	Betaxolol 2.5 MG/ML Ophthalmic Suspension (Betoptic S)	Ophthalmic suspension

^a^RxCUI: RxNorm Concept Unique Identifier.

Of the remaining 82 RxCUIs, 11 were RxCUIs corresponding to alpha-blockers primarily indicated for benign prostatic hyperplasia, 6 were RxCUIs for sacubitril/valsartan (Entresto) indicated for chronic heart failure with reduced ejection fraction, 1 was an RxCUI corresponding to an injectable antihypertensive product that is rarely used to treat HTN outside of a hypertensive crisis, and 1 was the *IN* term-type RxCUI for Potassium. A total of 63 were true antihypertensive drugs that were inadvertently omitted from the DrOn RxCUI map.

When the DrOn RxCUI Classification was compared with the Medication Name Classification using the DrOn RxCUI Classification as the reference, 695 terms overlapped, and 34 terms were unique to the DrOn RxCUI Classification (Figure 1). When the DrOn RxCUI Classification was compared with the Medication Name Classification using the Medication Name Classification as the reference, 195 terms overlapped, and 12 terms were unique to the Medication Name Classification. In total, 11 of the 12 terms that were unique to the Medication Name Classification were also included in the 135 terms that were unique to the RxClass ATC and MED-RT maps. The other term represented the brand name (Loniten) for the vasodilator minoxidil and was not present in any of the other classifications.

From the comparisons, the DrOn RxCUI Classification had 71 unique RxCUIs/terms that were not present in the RxClass MED-RT, RxClass ATC, and/or Medication Name classifications. Many were combination products (n=29), and the remaining 42 RxCUIs were spread across the other antihypertensive drug classes: ACE inhibitor (n=1), alpha-blocker (n=1), ARB (n=1), beta-blocker (n=17), CCB (n=11), loop diuretic (n=1), thiazide diuretic (n=3), and vasodilator (n=7).

Following the comparisons between the different drug classifications, 96 RxCUIs were added to the DrOn RxCUI Classification, and 15 antihypertensive medication names were added to the Medication Name Classification. The DrOn RxCUI Classification version 1.0 contains 2639 RxCUIs and is available in Multimedia Appendix 6, and the Medication Name Classification version 1.0 contains 301 antihypertensive medication names and is available in Multimedia Appendix 7 [10].

### Application of the Drug Classifications to OneFlorida

The results from applying the DrOn RxCUI Classification v1.0 and the Medication Name Classification v1.0 to the prescribing information from the OneFlorida HTN population are shown in Table 6.

Overall, the methods performed very similarly, with approximately 15% (2,080,685 versus 2,089,557/13,879,046) of the total prescriptions mapping to antihypertensive drugs. The DrOn RxCUI Classification v1.0 was able to map 8872 more prescription records to an antihypertensive drug class compared with the Medication Name Classification v1.0 (Table 6). When the different methods were compared by antihypertensive class, all classes were within 1% of each other (eg, 443,835/2,089,557, 21.24% vs 443,540/2,080,685, 21.32% of prescriptions mapped to the ACE inhibitor class using the DrOn RxCUI Classification method and Medication Name Classification method, respectively). Table 7 shows the specific antihypertensive class or classes each prescription was mapped to using the DrOn RxCUI Classification v1.0. The majority of the prescriptions mapped to a single antihypertensive class (eg, ACE inhibitors, beta-bockers; Table 7). However, 10.42% (217,682/2,089,557) mapped to combination antihypertensive products (Table 7).

**Table 6:** Summary of the methods to the OneFlorida Antihypertensive Prescribing Dataset.

Classification method	Antihypertensive (N=13,879,046)	
	Mapped, n	Coverage, %
Medication Name	2,080,685	14.99
DrOn^a^ RxCUI^b^	2,089,557	15.06

^a^DrOn: Drug Ontology.

^b^RxCUI: RxNorm Concept Unique Identifier.

**Table 7:** Application of the Drug Ontology RxNorm Concept Unique Identifier Classification to the OneFlorida antihypertensive prescribing dataset (N=2,089,557).

Antihypertensive Drug Class or Classes^a^	Frequency, n (%)
ACE^b^	443,835 (21.24)
Beta-blocker	411,721 (19.70)
Calcium Channel Blocker	360,653 (17.26)
Diuretic (Thiazide/Thiazide Like)	217,474 (10.41)
ARB^c^	178,252 (8.53)
Loop Diuretic	115,931 (5.55)
Diuretic/ACE Combo	92,275 (4.42)
Diuretic/ARB Combo	63,010 (3.02)
Centrally Acting	56,501 (2.70)
Vasodilator	30,665 (1.47)
Aldosterone Antagonist	29,214 (1.40)
Alpha Blocker	26,995 (1.29)

^a^Results are shown for antihypertensive drug classes that represent ≥1% of all antihypertensive prescriptions among the OneFlorida HTN population.

^b^ACE: angiotensin-converting enzyme inhibitor

^c^ARB: angiotensin II receptor inhibitor.

## Discussion

We created 2 different medication classification systems for antihypertensive drugs: one utilizing medication names and the other utilizing RxCUIs. After comparing these classification systems to each other, and to existing drug class terminologies available through RxNorm, we identified key areas that can lead to misclassification of antihypertensive medications and drug classes. Most misclassifications stemmed from failure to discriminate between dosage forms or issues related to primary indications of drugs (eg, selection of drugs that are primarily indicated for benign prostatic hyperplasia).

To illustrate, timolol is a beta-blocker that has both oral and ophthalmic dosage forms. The oral form is used to treat HTN, whereas the ophthalmic form is used to treat glaucoma [9,24]. An ideal antihypertensive drug classification system would include the oral form of timolol, but not the ophthalmic form. This is exactly what we see with the DrOn RxCUI Classification, as DrOn represents the therapeutic indication (HTN) for oral timolol separate from its MoA (beta-blocker) [8]. In contrast, all forms of timolol were included in our analysis that utilized the ATC and MED-RT terminologies. ATC only modeled the drug class, with all forms of timolol included as *beta-blocking agents*. MED-RT allowed for filtering through both a MoA relationship and a may-treat relationship. However, all forms of timolol, both oral and ophthalmic are mapped back to the ingredient term type for timolol, which has the may-treat relationship with HTN. Although this method allows for simplicity in mapping multiple drug products, it assigns an incorrect therapeutic indication (for our purposes) to the ophthalmic forms of timolol. We observed similar misclassification for other medications that have multiple clinical uses beyond HTN (eg, the vasodilator minoxidil and the alpha-1-blockers silodosin, alfuzosin, and tamsulosin). Minoxidil has oral dosage forms to treat HTN and topical foams to treat hair loss [25,26], whereas silodosin, alfuzosin, and tamsulosin are all alpha-1-blockers that are indicated for the treatment of benign prostatic hyperplasia as opposed to HTN [27,28].

Retired RxCUIs, or those that have been remapped to other classes, were classified by DrOn but not by MED-RT or ATC. This illustrates the need for maps and terminologies that include retired and obsolete RxCUIs as many of our longitudinal data sources include these. The data source used in this study contains data from January 2011 to July 2017, and contained 1170 RxCUIs that have been retired and 421 that have been remapped.

We conducted this work in the context of optimizing antihypertensive medication classification for use in HTN-related computable phenotypes. In other disease states where there are not as many options for pharmaceutical treatment, the classification of drug products may not be as complicated. However, in the case of HTN, and particularly the complex clinical phenotype of resistant hypertension (RHTN) [29-31], there are multiple classes of antihypertensive drugs with different mechanisms of action [30,32]. With RHTN, it is necessary to properly assign each antihypertensive medication to a medication class to determine if a patient meets the definition for RHTN, which is classically defined as requiring 4 or more antihypertensive drugs from different antihypertensive drug classes to achieve BP control [30,31]. This also illustrates the need to include a subject matter expert (eg, PharmD) during the creation of drug classification systems.

We also observed differences in the classification of combination drug products. There were some combination antihypertensive drug products that were not identified in the MED-RT terminology through the methods that we utilized in this study. Additionally, when utilizing a classification system based on medication name, all possible permutations of the combination name must be considered and included in the map (eg, HCTZ-metoprolol, hydrochlorothiazide-metoprolol, metoprolol-HCTZ, and metoprolol-hydrochlorothiazide). Without each of these permutations, it is possible to miss certain combination products. Finally, as a phenotype such as RHTN is determined partially, or fully, based on the antihypertensive drug count, a correct drug count must be assigned to each combination product. We have added this field into our DrOn RxCUI Classification v1.0.

Our study is not without limitation; currently, we do not have a gold standard antihypertensive medication classification list. We selected different data sources and compared classification based on these sources. However, we only used terminologies available through the US NLM. We have not included terminologies maintained by other groups (eg, American Hospital Formulary Service Pharmacologic-Therapeutic Classification) [33]. In addition, to classify based off of RxCUI, an RxCUI must be present. This may require additional work in some health care systems or datasets. We did not explore the temporal relationship between HTN diagnosis and antihypertensive medication prescription within our data. This could have resulted in the inclusion of medication data that was prescribed for indications other than HTN (eg, heart failure, atrial fibrillation, etc). We can examine this relationship more in our future work. Additionally, we did not explore the effect of the different methods and resources for creating the drug classification on the number of patients with RHTN within OneFlorida. We chose our study methodology because we first needed to create and validate this antihypertensive drug classification before we could use it in creating and validating our RHTN computable phenotype. Once our RHTN computable phenotype is validated, we can subsequently explore the effect of the different drug classification methods on the RHTN phenotype in future work. Other future work will include incorporating RxNorm Term Type (SCDF, SCD, SBD, etc), indicating retired RxCUIs, expanding to other disease states (diabetes, chronic kidney disease, heart failure, etc), and exploring the application to clinical decision support within the EHR [34,35].

In conclusion, we created and compared 4 different drug classification methods, focusing on the classification of antihypertensive drug products. We observed key differences in how each classification system handled drug products with multiple dosage forms, drug products with indications for benign prostatic hyperplasia, drug products that contain multiple antihypertensive ingredients (combination drug products), and RxCUIs that have been retired or remapped. We constructed 2 antihypertensive drug classification systems, 1 based off of RxCUIs and 1 based off of medication names. These are available for public use [10], and we will continue to update them through our own research.

## Appendix

Multimedia Appendix 1 Features of each Classification Method.

Multimedia Appendix 2 List of Anatomical Therapeutic Chemical relationships included as antihypertensive drugs.

Multimedia Appendix 3 List of Medication Reference Terminology has_MoA relationships included as antihypertensive drugs.

Multimedia Appendix 4 Initial Medication Name Classification.

Multimedia Appendix 5 Initial Drug Ontology RxNorm Concept Unique Identifier Classification.

Multimedia Appendix 6 Drug Ontology RxNorm Concept Unique Identifier Classification version 1.0.

Multimedia Appendix 7 Medication Name Classification version 1.0.

## Abbreviations

ACE: angiotensin-converting enzyme
API: application programming interface
ARB: angiotensin II receptor inhibitor
ATC: anatomical therapeutic chemical
BP: blood pressure
CCB: calcium channel blocker
CDM: common data model
DrOn: drug ontology
EHR: electronic health record
HTN: hypertension
ICD: International Classification of Diseases
MED-RT: Medication Reference Terminology
MoA: mechanism of action
NIH: National Institutes of Health
NLM: National Library of Medicine
PCORI: Patient-Centered Outcomes Research Institute
PCORnet: The National Patient Centered Clinical Research Network
RHTN: resistant hypertension
RxCUI: RxNorm Concept Unique Identifier
